# CGBayesNets: Conditional Gaussian Bayesian Network Learning and Inference with Mixed Discrete and Continuous Data

**DOI:** 10.1371/journal.pcbi.1003676

**Published:** 2014-06-12

**Authors:** Michael J. McGeachie, Hsun-Hsien Chang, Scott T. Weiss

**Affiliations:** 1 Channing Division of Network Medicine, Brigham and Women's Hospital, Boston, Massachusetts, United States of America; 2 Harvard Medical School, Boston, Massachusetts, United States of America; 3 Children's Hospital Informatics Program, Children's Hospital Boston, Boston, Massachusetts, United States of America; 4 Partners Health Care Center for Personalized Medicine, Boston, Massachusetts, United States of America; Carnegie Mellon University, United States of America

## Abstract

Bayesian Networks (BN) have been a popular predictive modeling formalism in bioinformatics, but their application in modern genomics has been slowed by an inability to cleanly handle domains with mixed discrete and continuous variables. Existing free BN software packages either discretize continuous variables, which can lead to information loss, or do not include inference routines, which makes prediction with the BN impossible. We present CGBayesNets, a BN package focused around prediction of a clinical phenotype from mixed discrete and continuous variables, which fills these gaps. CGBayesNets implements Bayesian likelihood and inference algorithms for the conditional Gaussian Bayesian network (CGBNs) formalism, one appropriate for predicting an outcome of interest from, e.g., multimodal genomic data. We provide four different network learning algorithms, each making a different tradeoff between computational cost and network likelihood. CGBayesNets provides a full suite of functions for model exploration and verification, including cross validation, bootstrapping, and AUC manipulation. We highlight several results obtained previously with CGBayesNets, including predictive models of wood properties from tree genomics, leukemia subtype classification from mixed genomic data, and robust prediction of intensive care unit mortality outcomes from metabolomic profiles. We also provide detailed example analysis on public metabolomic and gene expression datasets. CGBayesNets is implemented in MATLAB and available as MATLAB source code, under an Open Source license and anonymous download at http://www.cgbayesnets.com.

This is a *PLOS Computational Biology* Software Article

## Introduction

A Bayesian network (BN) is a data structure that encodes conditional probability distributions between variables of interest by using a graph composed of nodes and directed edges. In a BN, variables in the domain are modeled as random variables and represented by nodes, and edges between them represent a statistical dependence of the child node on the parent node. Each node is annotated with the conditional distribution of the variable given the values of its parents, and this information can be used to answer questions about the most probable values of variables in the BN given assignments to other variables in the BN.

BNs are attractive because they offer an interpretable picture of dependence and independence between domain variables, while modeling complex statistical relationships among them and providing prediction of an outcome of interest. BNs, as a mathematical modeling formalism, has enjoyed success in recent years [1] in predicting and modeling the genetic basis of complex disease, including stroke [2], nicotine dependence [3], and atherosclerosis [4]. In caricature, analysis by BNs can be broken into two steps: building the network (called network structure learning) and computing consequences of the network (called inference). Both steps are necessary in order to perform prediction of a disease phenotype in a biological dataset. First the network structure is learned from a case-control dataset comprising various potentially predictive biological or demographic variables, some of which will be included in the network. Then the parameters of this network are trained by looking at the conditional probabilities of the variables within the dataset. Finally, inference can be performed to predict the case or control status of new data points that contain measurements of the same variables.

However, Bayesian inference algorithms can be extremely complex and difficult to implement. There are several software packages for doing BN analysis, but to our knowledge all existing free implementations have one of two problems: 1) they do not allow mixed discrete and continuous data in a fully Bayesian mathematical formalism; or 2) they do not perform inference with the BN, merely performing the network learning step. The first limitation is one of traditional BNs, which are limited to considering only discrete variables. Researchers who wish to analyze continuous data (such as gene expression or metabolomics data) generally discretize these data, leading to loss of information and concurrent loss of power. In the second situation, a BN implementation that does not include inference is incapable of making predictions on new data, and researchers wishing to use the BN for prediction must either implement their own inference software, or take apart the BN model and put the pieces into some other modeling formalism, such as a logistic regression or a support vector machine. In addition to being inconvenient, this translation process leads to suboptimal performance since the variables identified in a BN are chosen for their relation and interactions to the other variables within the network – interactions which can be difficult to accurately recapitulate in another predictive modeling formalism.

CGBayesNets addresses both of these problems by implementing both network learning and network inference in a more modern type of Baysian network, the conditional Gaussian Bayesian network (CGBN) [5]. This is a network formalism wherein discrete and continuous nodes are mixed, with the stipulation that continuous nodes have Gaussian distributions linearly dependent upon any continuous parents with parameters conditioned upon the values of any discrete parents. In this formalism, discrete nodes cannot be modeled as statistically dependent upon continuous nodes, although the joint distribution can still be captured in the network. These are restrictions based on the available algorithms for performing exact Bayesian inference that we choose to implement [6], [7] (see also, “Design and Implementation”, below). Typically, these restrictions present little problem for the field of genomics, where we would generally model continuous gene expression values as being dependent upon discrete genetic polymorphisms – as in the case of expression quantitative trait loci (eQTL) analysis [8]. Indeed, integrative genomics applications of BNs have become increasingly attractive [9]. In a comparison study between BNs inferred from only expression data and BNs inferred from expression together with other types of genomic data, the combination of multiple genomic data types results in increased performance [10]. This agrees with our own experience: in our previous work using earlier versions of the CGBayesNets software we identified eQTLs in cancer datasets [11] and predicted leukemia types by integrating single nucleotide polymorphisms (SNPs) and messenger ribonucleic acid (mRNA) expression levels [8].

### Primary Analysis Scenario

CGBayesNets was primarily designed to aide genomic researchers in building predictive models of a phenotype of interest using multimodal genomic data, combined with demographic and clinical data, as possible predictors. A typical scenario is one where the researcher has a case-control dataset of patients with and without a binary condition: cancer [8], response to asthma steroid therapy [12], and survival time past a benchmark of clinical importance (28+ days after intensive care unit (ICU) admittance) [13], have been used in our previous work. CGBayesNets is used to construct a predictive BN model, which can then be used for predicting the phenotype of new data (e.g., predicting malignant vs. benign tumors from gene expression profiles; predicting which asthmatics responds to inhaled corticosteroids therapy; or predicting ICU prognosis from metabolomic profiles). To employ CGBayesNets in these cases, a dataset of all the possibly predictive variables – SNPs, gene expression measurements, demographic data – along with the phenotype (case/control status), are combined for each subject; this is provided to CGBayesNets. Using CGBayesNets, the researcher can then apply one of several network learning algorithms to find a network predictive of the phenotype. This network may include many or few connections to the phenotype, where variables that are unconnected are not necessary for prediction of the phenotype. After a satisfactory Bayesian network is obtained, the CGBayesNets inference routine can predict the outcome (case/control status) of each patient; in this way the predictive accuracy of the network can be assessed. To provide the best validation of the network, the network can then be used on a replication dataset, one that contains the same variables and outcome as used in the network, and prediction accuracy on a new dataset can be assessed.

Of course, the primary outcome of a clinical case-control trial need not be used; other discrete variables of interest can be considered the phenotype; and networks predictive of these secondary outcomes can be computed separately. In a future update, CGBayesNets will also allow continuous (normally-distributed) variables to be used as the phenotype.

### Related Software

While there are several software packages for learning and predicting with Baysian networks, none provide the mix of features presented by CGBayesNets; in particular no free implementations provide algorithms for inference in networks of mixed discrete and continuous variables.

Some Bayesian packages focus only on learning the network structure, including the popular Banjo [14] package. This package includes several methods for learning both dynamic and static BNs, although none for inference with those networks. It does not include a Bayesian treatment of continuous variables and requires those to be discretized to be included in the models. Other software for learning Bayesian networks do treat continuous variables with full Bayesian semantics but do not implement inference for such models. These include the DEAL [15] and BNLearn [16] packages in the R statistical language.

Other BN packages provide network learning and inference but do not implement inference with continuous variables without discretization. These include the machine learning Java platform Weka 3.6.9 [17] and the GeNIe and Smile packages (Decision Systems Laboratory, University of Pittsburgh, http://genie.sis.pitt.edu/index.php). Other large and comprehensive packages are commercial, which limits their availability and availability of the source code, and thus cannot be easily integrated into researcher's software, although they may provide full-featured BN processing (e.g., Hugin Expert – Hugin, Aalborg, Denmark. http://www.hugin.com).

Some network-learning packages are not Bayesian, but instead use other formalisms for defining statistical relationships between variables, such as GlobalMIT [18], which uses mutual information to learn a dynamic (non-Bayesian) network. Also in this category is the Uninet package which relies on conditional rank correlation to define statistical dependence between variables [19].

GDAGSim is a Gaussian linear model simulator [20], which allows users of the C library to define and perform statistical inference and simulation on network models of normally distributed quantities. This is of potential interest to those wishing to simulate purely continuous Bayesian networks, but does not implement the integration of discrete nodes to implement CGBNs; nor does it include any structure learning.

Perhaps closest to our CGBayesNets package is the BNfinder 2.0 package [21], which focuses on learning dynamic BNs from time-series data, and includes routines that select the most likely child value for a node given the values of that node's parents, rather than full inference and prediction. Dynamic BNs are BNs that include a time constraint indicating that some nodes come before other nodes temporally (as in time-series experiments), or causally (as in metabolic pathways). Learning optimal dynamic BNs is computationally easier than learning optimal traditional (static) BNs [22], but BNfinder 2.0 can learn static BNs given K2-style node-ordering constraints. BNfinder 2.0 does allow continuous variables but only allows them to have exactly one discrete parent and zero children, which limits some of the possible networks that can be modeled.

In total, CGBayesNets provides new functionality: learning and predicting with Bayesian networks composed of discrete and continuous variables. In the following, we discuss our implementation of these and additional features.

## Design and Implementation

CGBayesNets is entirely Bayesian, using the Bayesian marginal likelihood to guide network search and for performing inference. Using Bayesian statistics allows leveraging of Bayesian priors to bias network structure learning toward parsimonious models that are more likely to predict well on new datasets, while also providing a consistent mathematical treatment throughout the package. Please see the Supplementary Materials for a full mathematical treatment of the Bayesian semantics.

### Inference Algorithms

We refer to the process of predicting an outcome of interest using a Bayesian network as “inference,” the term commonly used for this in the BN literature. Inference in a BN can proceed either forward or backward along the directed edges of the network. The best prediction of a phenotype node is often obtained when that node has no parents, but many children. This is structurally similar to a type of network know as a Naïve Bayes Network, where each other (non-phenotype) node is conditionally independent given the phenotype. Although Naïve Bayes networks are simple, in practice they can provide extremely good prediction [23]. The opposite network structure – a phenotype node with many parents and no children – frequently results in very poor predictive performance, because a child node's distribution is conditional on the combination of values of each of its parents. As the number of parents of a node increases, the number of parameters describing the distribution of the child increases exponentially. For practical datasets, this can result in very few datapoints of each combination of parent values from which to estimate the phenotype's conditional distribution; a problem referred to as data fragmentation. This leads to poor estimation of the phenotype's distribution, and this in turn leads to poor prediction. When accurate prediction is required, it is thus expedient to obtain networks where a phenotype has many child nodes and no parent nodes – a heuristic employed in CGBayesNets.

To perform inference in a CGBN, different algorithms are used on the discrete and continuous portions of the network. We have implemented the Cowell algorithm for inference in conditional Gaussian network nodes [24], and combine that algorithm with a simple variable elimination algorithm for inference between discrete nodes in the network [25].

We chose the Cowell algorithm for inference because it is numerically stable. There are very few algorithms to choose from for exact Bayesian inference in mixed networks. The Cowell algorithm is based on the earlier Lauritzen and Jensen junction tree algorithm [26]. Both of these are an improvement over the original algorithms for inference in CGBNs [27] that were found to be numerically unstable due to repeated matrix inversions. The discrete inference algorithm is a typical variable elimination algorithm, as described in Koller and Friedman [25]. The algorithm involves creating factors that represent groups of nodes in the discrete part of the CGBN, and implementing the factor product and sum operations upon these to compute either marginal or conditional distributions of a node given the value of parents or children. For details, see chapter 9, Koller and Friedman [25].

### Network Learning Algorithms

While the focus of CGBayesNets is on inference in mixed Bayesian networks, CGBayesNets provides four main network structure search algorithms. The problem of searching for the best Bayesian network is one that has received much attention over the last 30 years, and there are many possible algorithms (and software implementations of those) that a researcher may want to employ. We have endeavored to make our software package modular and extensible so that researchers familiar with MATLAB will be able to easily add their own network search algorithms; we also read common network file formats so that researchers can use other packages that have more extensive and specialized network search procedures to find a good Bayesian network, and then use CGBayesNets to perform inference in that network.

In all of the network search algorithms in CGBayesNets, network scoring is done by a metric sometimes known as the *Bayesian Dirichlet equivalent sample-size, uniform (BDeu)*, which is a measure of the marginal likelihood of the data, given the network [28], (see also supplemental material). In every algorithm, CGBayesNets looks for the network that maximizes the marginal likelihood of the data. Although this is not necessarily the network with the best phenotype prediction, it is the network that is best supported by the available evidence – the data. Since the number of possible networks is super-exponential in the number of variables (for n variables, there are ∼2∧(n^2^) possible directed networks), all networks cannot be investigated; rather heuristics are employed to search for good networks that might be satisfactory. One heuristic we employ throughout is a limit on the maximum number of possible parents a node may have, which may be set by the researcher to any appropriate value.

The first network learning algorithm we provide in CGBayesNets is a K2-style [29] search that orders nodes by likelihood of statistical independence, such that nodes so ordered can have parents only occurring before them in the order, and that the nodes most likely to be independent are given the fewest possible parents. The main benefit of such a list is that cycles are impossible, and this is important for learning BNs – which must be acyclic. We then learn parents of each continuous node according to this order, using a stepwise adjustment that allows the addition of one parent to a node to trigger the removal of an existing parent if that increases likelihood. Next we learn parents of every discrete node, in a similar way. The K2 algorithm is very fast, considering only k/2 * n^2^ possible edges, where n is the number of variables and k is the maximum number of parents a node can have, a parameter of the search algorithm. K2 is frequently almost as good as other methods that consider a much larger number of possible edges, and thus take much more computational time. The K2 procedure we have implemented can be considered a hill-climbing algorithm that allows backtracking, but does not consider all possible edges, only those that obey the K2 ordering constraint.

A second structure learning algorithm provided in CGBayesNets is a greedy, exhaustive, search algorithm that starts with an empty network and adds the best edge, iteratively. It does not rely upon a K2-style node priority list to avoid cycles, but rather does its own cycle-checking with depth-first search. This algorithm is a greedy hill-climber, in that at every step it adds the edge that increases data likelihood the most. It is exhaustive in that it considers all possible legal edges, between any two nodes (not that it considers all possible *networks*). It may be run with or without allowing backtracking, which if enabled will also consider the removal of any existing edge, if that removal results in the greatest increase in likelihood. This algorithm is more comprehensive than the K2 algorithm – it considers potentially n^2^+kn^2^ edges, and may find better networks than K2, although it is much slower. In sum, this algorithm is appropriate for smaller datasets, and will have prohibitive computational cost for networks with thousands of nodes.

The third network learning algorithm provided with CGBayesNets is pheno-centric search, which builds a network based around a particular phenotype node, sufficient to perform learning and prediction of that particular variable [8]. The Markov properties of the BN semantics state that the distribution of a node is conditionally independent given the values of its parents, children, and other parents of those children (collectively, the “Markov blanket” or “Markov neighborhood” of the node). Only these nodes are required to predict the value of a phenotype. Thus, if we are only interested in predicting the value of a phenotype node, we need not build the entire BN over all of the variables in the domain; we only need to look for its Markov blanket. Such a pheno-centric network has two main benefits. First, it can allow prediction in domains where building a full BN over all the variables is computationally prohibitive, as a pheno-centric search considers at most n+kn^2^ possible edges. Second, it does not require a K2-style node order list for parent constraints, and as such does not needlessly exclude many potential network structures from consideration. On the other hand, it can result in overfitting of the data: making too many connections to the phenotype that may just be due to random noise in the dataset.

The fourth algorithm is a hill-climbing technique known as simulated annealing [30], which is very similar to our second algorithm (the greedy, exhaustive hill-climber). Simulated annealing search initially will add *any randomly-chosen edge* to a network, rather than the best edge. As the search goes on, the probability of adding edges that reduce, rather than increase, likelihood decreases, slowly, to zero. This method has the benefit of being able to run in however much time the researcher may have; and providing solutions of increasing likelihood given increasing computational time, indeed we recommend considering n^3^ possible edges to allow the search to consider many possible permutations of the n^2^ possible edges in a network. However this is the slowest of our four search algorithms and as such may perform worse than the other three given limited computational time.

Finally, for learning networks of many variables, CGBayesNets includes simple filtering functions that filter the number of variables by Bayes Factor of association with the phenotype, where the Bayes Factor is the ratio of posterior likelihood of the data with the variable dependent upon the phenotype, to the likelihood of the data independent of the phenotype [31]. Such filtering strategies are necessary for pruning a dataset of many thousands of variables down to a smaller set of informative variables for BN analysis.

### Software Features

The CGBayesNets package is intended to support all phases of the predictive modeling process.

CGBayesNets provides the four network structure learning algorithms, described above. In addition, in our software implementation, CGBayesNets provides separate functions for learning the parameters of a network and learning its structure from data, and base functions for computing Bayesian likelihood of variables. These functions make it easy for advanced users to add their own network learning algorithms. Once structure and parameters are learned, the model may be tested on a dataset: either the existing dataset or a new (replication) dataset. CGBayesNets provides functions for making testing on multiple different datasets simple and direct. In all cases the Area Under the Receiver-Operator Characteristic Curve (AUC) is reported as a measure of predictive accuracy of the network [32]. This is provided with its convex-hull AUC and 95% confidence intervals, together with functions for computing p-values for difference between two AUCs executed over the same dataset, using the method of Delong *et al.* [33].

To increase the performance of networks on replication datasets, CGBayesNets provides functions for employing cross-validation (CV) and bootstrapping. The cross-validation functions will either perform CV to determine the best settings of Bayesian prior parameters, or to estimate the performance on an unknown replication dataset. Bootstrapping is provided to obtain estimates of the frequency of individual edges within a given Bayesian network, by comparing frequencies of edges in different bootstrap realizations of the dataset. This results in a single aggregate network with fractional probabilities for each edge; functions are provided to translate these into concrete Bayesian networks and test their performance.

We have endeavored to make CGBayesNets easier to use by providing several data reading and writing functions. There are input functions for reading several different types of PED SNP files, and text files formatted with mixed string and numeric data, such as output by the popular R statistical language. We output networks into Trivial Graph Format (tgf), which can be manipulated for example by the free program yEd (yWorks, Tubingen, Germany. http://www.yworks.com/en/products_yed_about.html), or the SIF and GraphML formats for use with the program Cytoscape [34].

CGBayesNets is distributed as MATLAB source code. Each function is commented and documented with the input and output specifications so that it may be employed in the user's application as necessary. To make this as easy as possible, we make recommendations as to which functions are suggested for modification, and which represent inner workings of the algorithms, and should not generally be altered. We also provide example code to copy and edit demonstrating how to combine our lower-level Bayesian inference functions to assemble higher-level search and diagnostic routines.

## Results

### Results from Biological Applications

The primary form of biological insight provided by CGBayesNets is predictive network models that differentiate cases from controls. CGBayesNets is the only existing free software package for doing so with Bayesian networks of mixed discrete and continuous domains.

It is clear that discretization of continuous variables is a possibility, allowing researchers to convert continuous variables to discrete ones and then use discrete Bayesian network methods. However, we argue that this necessarily results in a loss of information and a concomitant loss in power. See supplemental material for an example of a mixed discrete-continuous domain where we compare performance of BNs using discretization of continuous variables to using CGBayesNets. Results from this experiment are shown in Table 1, and the difference between discretized performance (72.6% AUC) and the original performance (99.3% AUC) is considerable.

**Table 1:** Performance of various classifiers on a simple example dataset, discretization/data_gen2_train.csv and discretization/data_gen2_test.csv, trained and tested on separate data generated from a BN of 5 discrete and 15 continuous variables (n = 25), and also averages over 10 similar, randomly generated networks.

	True Network	BNfinder 2.0 (K2)		BNfinder 2.0 (reverse-K2)		Weka 3.6.9		CGBayesNets	
Data	nodes	Nodes	AUC	Nodes	AUC	nodes	AUC	nodes	AUC
Original	14	5	50%	3	98.7%	-	-	8	99.3%
Discretized	14	3	50%	2	61.3%	20	50.0%	5	72.6%
Average Original	-	-	68.7%	-	82.4%	-	-	-	99.2%
Average Discretized	-	-	68.7%	-	68.7%	-	70.0%	-	70.0%

This illustrates the differences between CGBayesNets and two other software packages, BNfinder 2.0 and Weka 3.6.9. Training data is presented in two forms: in its original form, and in its discretized form, where continuous nodes are binned into 10 equal-width discrete bins. The “nodes” columns refer to the number of nodes in the Markov blanket of the phenotype node in the discovered network. “AUC” is the Area Under receiver-operator characteristic Curve, evaluated by predicting the phenotype node given values of the other 19 nodes in a separate test set. “BNfinder 2.0 (K2)” refers to BNfinder 2.0 supplied with node-parent constraints consistent with the topological ordering of nodes in the true network. “BNfinder 2.0 (reverse-K2)” indicates BNfinder 2.0 supplied with node-parent constraints consistent with the opposite of the topological ordering of the nodes in the true network. No data is reported for Weka 3.6.9 on the Original data, since Weka 3.6.9 does not handle Bayesian Networks with continuous data variables. Average AUCs over 10 similarly-generated networks are shown for each method, before and after discretization.

We have used CGBayesNets in several applications. We have performed eQTL analysis with earlier versions of the software that resulted in predictive models for subtypes of acute lymphoblastic leukemia [8] with the gene expression and gene variation (SNP) data available with Gene Expression Omnibus (GEO) accession #GSE10792 [35]. Other groups have used CGBayesNets, and these have resulted in predictive models for tree and wood characteristics [36] from mixed gene expression and SNP datasets. This application used the cross-validation routines in CGBayesNets to identify highly-predictive subsets of variables for different tree phenotypes, and then to prompt further biological analysis of these predictors.

To consider one application in greater detail, we recount the network analysis strategy employed in our previous work: the identification of a metabolic signature for predicting mortality in hospital intensive-care units (ICU) from metabolomic profiling [13]. We started with a sample of 187 biological metabolites from 90 ICU patients together with clinical and demographic data including age, sex, renal function, and APACHE II score (an aggregate score indicative of ICU prognosis). We used 5-fold cross-validation on the training data to arrive at hyper parameters for the Bayesian likelihood calculations. We performed 2500 bootstrap realizations of the training dataset, and learned a pheno-centric CGBN for each bootstrap realization. From the sample of 2500 networks, we built a consensus network by starting with the phenotype node and then adding, in sequence, the most frequent edge occurring in the bootstrap networks, and measuring the performance of that network on the dataset in cross-validation. This provided a way of estimating the value of adding each node to the network, and roughly the point of diminishing returns. We used a network with a total of seven predictive nodes to define the final network model, as adding further nodes did not increase the predictive performance in cross-validation. The final network of seven metabolites achieved 91% AUC for predicting mortality in the training dataset, and we validated that network in an independent replication population of 120 ICU patients from a separate cohort, obtaining an AUC of 74% - significant prediction despite significant clinical (cancer rates) and demographic (Caucasian vs. African American) differences in the training and testing cohorts [13]. The seven metabolites identified (gamma-glutamylphenylalanine, gamma-glutamyltyrosine, 1-arachidonoylglycerophosphocholine* (20∶4), taurochenodeoxycholate, 3-(4-hydroxyphenyl)lactate, sucrose, and kynurenine) are potentially employable as an ICU outcome prediction tool in future clinical settings.

### Results on Test Data

We include several test datasets with the CGBayesNets download. These are intended both to demonstrate the suggested use of our software and to assure its correct installation and function. We provide a metabolomic profiling dataset of a cachexia sample from the MetaboAnalyst2.0 [37] website (http://www.metaboanalyst.ca/MetaboAnalyst/faces/Home.jsp ‘human_cachexia.csv’). The suggested model achieves 86.8% AUC in cross-validation. We then discuss performance of CGBayesNets on a differential gene-expression dataset from GEO, accession #GSE19301, as described by Bjornsdottir et al. [38]. This identifies models using a training subset of the dataset that are predictive of the testing subset of the dataset using several transcripts with strong linear effects. Full details of how to compute these results are given in the supplemental materials.

## Availability and Future Directions

Bayesian Networks remain an important machine learning methodology within bioinformatics, although their recent application in genomics, metabolomics, and proteomics has been limited by the necessity to learn networks over mixed discrete and continuous variables. The development of effective algorithms for learning and reasoning with conditional Gaussian Bayesian networks addresses this issue, although freely available implementations of these algorithms have so far been unknown. Our CGBayesNets package solves this problem and fills these needs. We are committed to continued development of CGBayesNets to fit our own needs of predictive Bayesian network software, as we continue to apply these techniques to biomedical domains; these improvements will be available to all users of CGBayesNets in the future.

The CGBayesNets package is available from the authors and via anonymous download from www.cgbayesnets.com. CGBayesNets is open source software, and is distributed as MATLAB source. It has been verified to run on both Linux and Windows platforms.

## Supporting Information

Figure S1 **Bayesian Networks of Cachexia.** These networks are formed by running CGBayesNets bootstrapping routine on the human cachexia dataset from (http://www.metaboanalyst.ca/MetaboAnalyst/faces/Home.jsp ‘human_cachexia.csv’). Each network shows the Markov blanket of the phenotype of interest (“Muscle loss”), which are those nodes necessary to predict muscle loss. Arrows between nodes indicate statistical dependence of the child node on the parent node(s), and do not represent causality. Networks are learned on 25 bootstrap realizations of the data, and those shown are consensus networks including the two (a), three (b), four (c), and eight (d) most frequently included edges in the bootstrap networks. Performance of these networks is given in Table S1 in Text S1. Images generated and formatted with the yEd program (yWorks).
(PNG)

Figure S2 **Comparison of four different network search algorithms on training and test data.** Training performance is measured with five-fold cross-validation. For each of four search algorithms (K2, Pheno-Centric, Full-Exhaustive, and Naïve-Bayes) 5 bootstrap realizations of the training data were generated and a Bayesian network was learned for that realization. The x-axis represents using the most frequent N edges occurring in the population of bootstrap networks to create a consensus Bayesian network of at least N edges.
(TIFF)

Figure S3 **Comparison of prediction performance with continuous features vs. discretized features.** This graph shows the difference in predictive performance (measured by change in AUC predicting the phenotype node from training dataset to testing dataset) in a dataset including continuous variables and the same dataset after continuous datasets were discretized into 10 equal-sized bins. Each circle represents a different random network created on 25 nodes, each randomly chosen to be discrete or continuous. The size of the circle is proportional to the number of datapoints simulated from that network, N, ranging from 25 at the smallest circles to 200 at the largest circles. Experiments are ordered by increasing sample size (N), along the x-axis. The color of the circle represents the number of nodes in the Markov blanket of, and therefore required for prediction of, the phenotype node. The red line represents a regression of difference in predictive performance on the x-axis. This regression indicates that when the number of variables is similar to the sample size, performance is on average 13% worse after discretizing continuous variables; while the difference goes away when sample size far exceeds the number of variables. Experiments where there is no difference between continuous performance and discretized performance not included in this analysis.
(PNG)

Software S1 **This zip file contains CGBayesNets.** It is distributed as MATLAB script files along with supporting data files, referenced in the text and in the Supporting Text S1.
(ZIP)

Text S1 **This file contains additional text and material pertaining to issues raised in the main body.** Supplemental Sections: Section 1: CGBayesNets Installation. Section 2: Example Analysis, Narrative Analysis in Metabolomics. Section 3: Example Analysis, Code Examples with Gene Expression Data. Section 4: Illustrative Discretization Example. Section 5: Theoretical Foundations.
(DOCX)
