# Supplementary figures and images for: CGBayesNets: Conditional Gaussian Bayesian Network Learning and Inference with Mixed Discrete and Continuous Data
Source: PLoS Comput Biol. 2014 Jun 12;10(6):e1003676. doi: 10.1371/journal.pcbi.1003676 (PMC4055564; doi:10.1371/journal.pcbi.1003676)

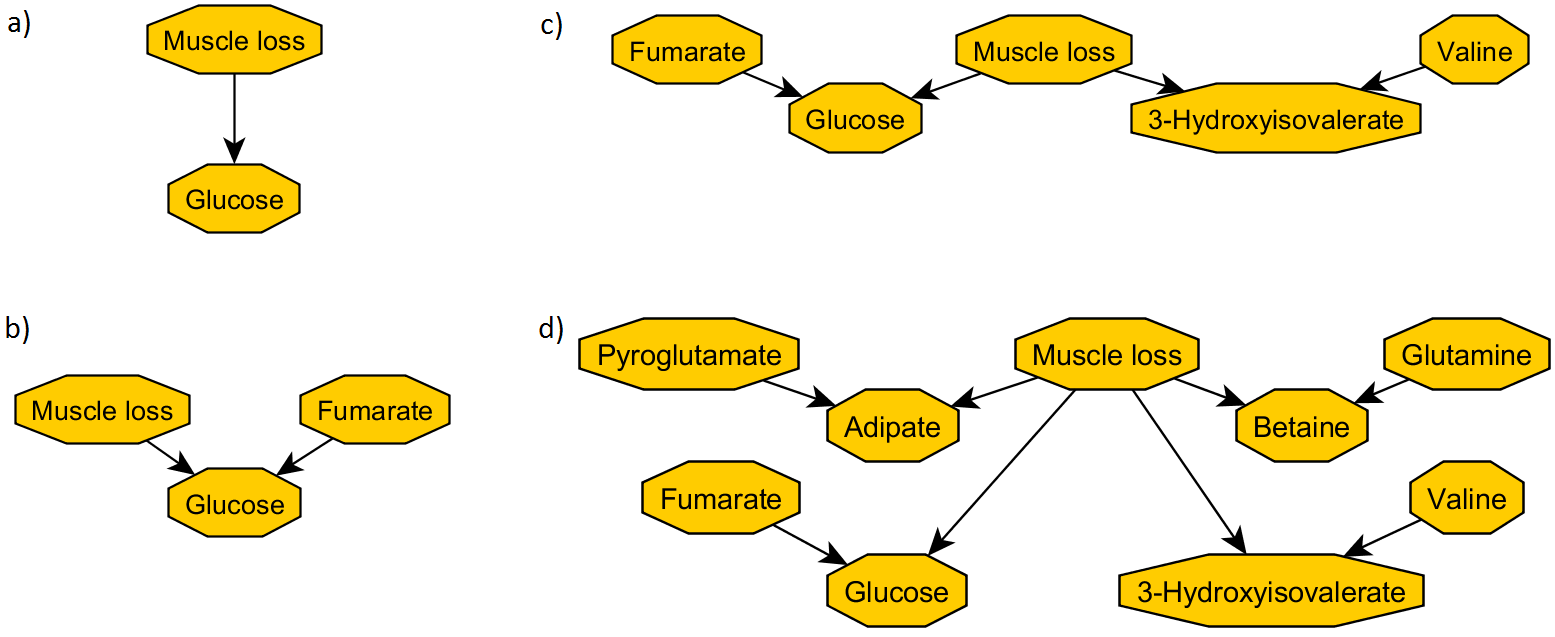

Supplement: Figure S1 — Bayesian Networks of Cachexia. These networks are formed by running CGBayesNets bootstrapping routine on the human cachexia dataset from (http://www.metaboanalyst.ca/MetaboAnalyst/faces/Home.jsp ‘human_cachexia.csv’). Each network shows the Markov blanket of the phenotype of interest (“Muscle loss”), which are those nodes necessary to predict muscle loss. Arrows between nodes indicate statistical dependence of the child node on the parent node(s), and do not represent causality. Networks are learned on 25 bootstrap realizations of the data, and those shown are consensus networks including the two (a), three (b), four (c), and eight (d) most frequently included edges in the bootstrap networks. Performance of these networks is given in Table S1 in Text S1. Images generated and formatted with the yEd program (yWorks). (PNG) [file pcbi.1003676.s001.png]

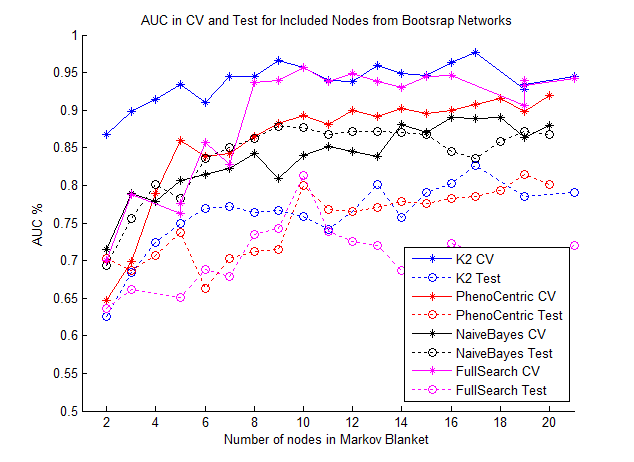

Supplement: Figure S2 — Comparison of four different network search algorithms on training and test data. Training performance is measured with five-fold cross-validation. For each of four search algorithms (K2, Pheno-Centric, Full-Exhaustive, and Naïve-Bayes) 5 bootstrap realizations of the training data were generated and a Bayesian network was learned for that realization. The x-axis represents using the most frequent N edges occurring in the population of bootstrap networks to create a consensus Bayesian network of at least N edges. (TIFF) [file pcbi.1003676.s002.tif]

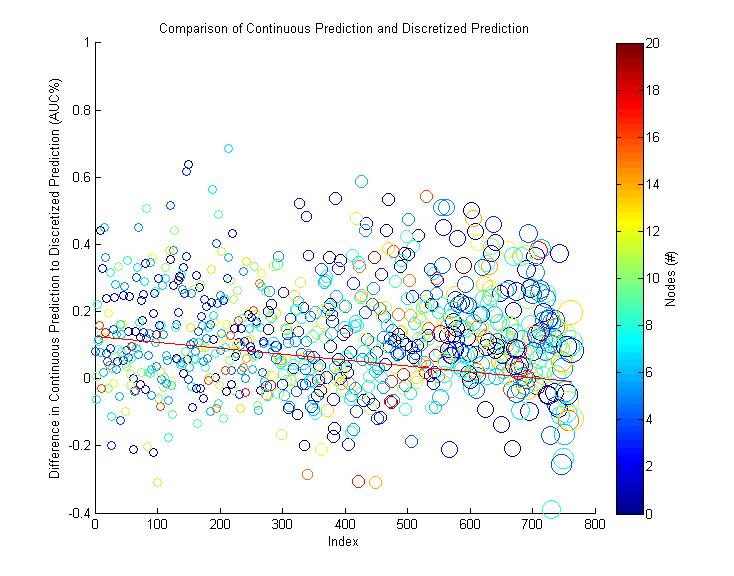

Supplement: Figure S3 — Comparison of prediction performance with continuous features vs. discretized features. This graph shows the difference in predictive performance (measured by change in AUC predicting the phenotype node from training dataset to testing dataset) in a dataset including continuous variables and the same dataset after continuous datasets were discretized into 10 equal-sized bins. Each circle represents a different random network created on 25 nodes, each randomly chosen to be discrete or continuous. The size of the circle is proportional to the number of datapoints simulated from that network, N, ranging from 25 at the smallest circles to 200 at the largest circles. Experiments are ordered by increasing sample size (N), along the x-axis. The color of the circle represents the number of nodes in the Markov blanket of, and therefore required for prediction of, the phenotype node. The red line represents a regression of difference in predictive performance on the x-axis. This regression indicates that when the number of variables is similar to the sample size, performance is on average 13% worse after discretizing continuous variables; while the difference goes away when sample size far exceeds the number of variables. Experiments where there is no difference between continuous performance and discretized performance not included in this analysis. (PNG) [file pcbi.1003676.s003.png]
