# Supplementary material for: CGBayesNets: Conditional Gaussian Bayesian Network Learning and Inference with Mixed Discrete and Continuous Data
Source: PLoS Comput Biol. 2014 Jun 12;10(6):e1003676. doi: 10.1371/journal.pcbi.1003676 (PMC4055564; doi:10.1371/journal.pcbi.1003676)
Supplement: Text S1 — This file contains additional text and material pertaining to issues raised in the main body. Supplemental Sections: Section 1: CGBayesNets Installation. Section 2: Example Analysis, Narrative Analysis in Metabolomics. Section 3: Example Analysis, Code Examples with Gene Expression Data. Section 4: Illustrative Discretization Example. Section 5: Theoretical Foundations. (DOCX) [file pcbi.1003676.s005.docx]

CGBayesNets: Conditional Gaussian Bayesian Network Learning and Inference with Mixed Discrete and Continuous Data

Michael J. McGeachie^1,2,*^,

Hsun-Hsien Chang^2,3,*^

Scott T. Weiss^1,2,4^

^1^Channing Division of Network Medicine, Brigham and Women’s Hospital, Boston, MA, USA.

^2^Harvard Medical School, Boston, MA, USA.

^3^Children’s Hospital Informatics Program, Children’s Hospital Boston, Boston, MA, USA.

^4^Partners Health Care Center for Personalized Medicine, Boston, MA, USA

*These authors contributed equally to this work.

Correspondence to: Michael McGeachie, [michael.mcgeachie@channing.harvard.edu](mailto:michael.mcgeachie@channing.harvard.edu)

Supplemental Materials.

Supplemental Sections:

Section 1: CGBayesNets Installation.

Section 2: Example Analysis, Narrative Analysis in Metabolomics.

Section 3: Example Analysis, Code Examples with Gene Expression Data.

Section 4: Illustrative Discretization Example.

Section 5: Theoretical Foundations.

**Section 1. CGBayesNets Installation.**

CGBayesNets is a MATLAB API for building, discovering, and predicting with conditional Gaussian Bayesian networks. It requires a recent version of MATLAB to run, and we suggest something more recent than MATLAB version 2011b, which contains a bug that makes CGBayesNets unusable. CGBayesNets has been tested on both Linux and Windows and provides identical results. No MATLAB toolboxes are required.

Unzip the CGBayesNets zip file into its own directory, perhaps /cgbn/. Start up MATLAB. From within MATLAB, navigate to /cgbn/, and then run the bnpathscript.m file. This can be done with commands:

>> cd /cgbn/

>> bnpathscript;

This will update the MATLAB path to include the subdirectories of /cgbn/ necessary for CGBayesNets to function. This script will have to be run every time MATLAB is restarted; alternatively, you may permanently add the subdirectories of /cgbn/ to the MATLAB path using the MATLAB interface.

**Section 2. Example Analysis: Narrative Analysis in Metabolomics**

To demonstrate analysis of biological data with CGBayesNets, we applied our software to the human cachexia metabolomics dataset available at the MetaboAnalyst2.0 [[1](#_ENREF_1)] website (<http://www.metaboanalyst.ca/MetaboAnalyst/faces/Home.jsp> ‘human_cachexia.csv’). This is a dataset with 77 patients with continuous measurements for each of 63 different biological metabolites, and including 47 cases of cachexia and 30 controls.

We first loaded the data and log2-transformed each of the metabolite measurements. Metabolite measurements were then normalized. The simplest analysis can be easily accomplished by first calling the LearnStructure() function to learn the network structure of the BN maximizing the posterior likelihood of the data. Next the LearnParams() function can be called to learn the marginal distributions of each node in the BN based on the dataset and the Bayesian priors. Finally, PredictPheno() can be used to predict the phenotype variable, “Muscle loss”, and returns the AUC of the BN, together with its convex-hull AUC and 95% confidence intervals.

However, for this example, we use a more interesting approach and first learn the network using the bootstrapping functionality. We call the BootstrapLearn() function to generate BNs for 250 bootstrap realizations of the dataset. With the resulting adjacency matrix of edge frequencies, we then used the BootsNetAddEdgesCV() function to compare the performance of networks formed by starting with the phenotype node, and then adding, in sequence, the most frequent edge occurring in the bootstrap networks, and measuring the performance of that network on the dataset in cross-validation. This function provides a way of estimating the value of adding each node to the network, and roughly the point of diminishing returns. We consider four different networks, formed by adding the first two (Figure S1a), three (Figure S1b), four (Figure S1c), and eight (Figure S1d) most-frequently included edges in the bootstrap realizations. These networks are obtained from the NEdgesFromBoots() function, the marginal distributions instantiated from the dataset with LearnParams(), and the predictive performance assessed with PredictPheno(). Each of these networks is shown in Figure S1, with its associated convex-hull AUC values in Table 2. As a general principal of predictive model building, one should prefer the most parsimonious model (that is, the smallest model) among models with equal (or similar) performance. Our AUC comparison functions provided p-values for difference between each of the four networks’ performance (using function CompTwoAUC(), which implements the method of DeLong *et al.* [[2](#_ENREF_2)]), which indicated that there is a significant difference between model D and model B, and nearly significant improvement over model A. We therefore suggest that the 8-node model (Figure S1d) provides a significant improvement in model performance, and is the most likely to display good performance on similar, unseen data. All the analysis for human_cachexia.csv are provided with the CGBayesNets package in the file data/testdata/AnalyzeCachexia.m.

**Section 3. Example Analysis: Code Examples with Gene Expression Data**

To demonstrate analysis of gene expression data with CGBayesNets, we applied our software to the Gene Expression Omnibus (GEO) dataset #GSE19301, as described by Bjornsdottir et al.[[3](#_ENREF_3)] This is a dataset of 118 asthma patients who experience asthma exacerbations, and have peripheral blood drawn both concurrent with the exacerbation and at up to five time points other than the exacerbation. From each of these samples, complete gene expression data is assessed using a Affymetrix Human Genome U133A Array (HG-U133A; Affymetrix, Santa Clara, CA, USA.)

These data were obtained from GEO, and preprocessed as follows. The original datafile was split into two datafiles that are easier for MATLAB to process: one representing the gene expression data for each sample (rows are samples, columns are mRNA expression probes); and a second representing the phenotypic and demographic data. These are the GSE19301_pheno_matrix_simple.txt and GSE19301_data_matrix.txt files, found in the /cgbn/data/testdata/GEO/ directory.

1) Start MATLAB. Navigate to /cgbn/ and run:

>> bnpathscript;

2) navigate to /cgbn/data/testdata/GEO and run:

>> ProcessGeoSet();

This does three main things: 1) remove unnecessary data columns; 2) use AffyGeneMap_GPL96-15653_simple.txt to rename mRNA probes with the canonical gene names; 3) average the non-exacerbation samples per individual, and then subtract this from the one exacerbation sample to get a differential expression measurement. This outputs a file GSE19301_diffexpr_geneids.txt that is then used in further analysis.

Further analysis of this dataset proceeds with the TestDiffEx.m file. This is a script file that contains commands for loading GSE19301_diffexpr_geneids.txt, and performing various different Bayesian Network analyses. This script file can be invoked directly, although this will take time to run; or it can be executed a few commands at a time. MATLAB script files can be divided into code blocks by lines starting with double comment characters, ‘%%’, and opening the script file in the MATLAB editor and putting the cursor in a code block and then pressing CTRL-Enter will execute that block of code only.

Block 1 loads the differential expression data, drops some unwanted columns, and normalizes continuous-valued variables.

fprintf(1,'Loading and processing GSE190301 differential expression data\n');

[data, cols, strdata, dcolinds, scolinds] = RCSVLoad('GSE19301_diffexpr_geneids.txt',false,'\t');

% make exacerbation under greater steroid exposure the phenotype:

pheno = 'maximum steroid exposure 4=systemic 3=inhaled 2=intranasal 1';

dropcols = {'Exacerbation','Donor','leukotriene receptor antagonist', ...

'baseline severity nih guideline'};

drops = zeros(size(cols));

for i = 1:length(dropcols)

drops = drops | strcmpi(dropcols(i),cols);

end

% drop the columns we're not interested in

cols = cols(~drops);

data = data(:,~drops);

% move phenotype to column #1

phenocol = strcmpi(pheno,cols);

data = [data(:,phenocol),data(:,~phenocol)];

% and rename phenotype:

pheno = 'Steroid Exacerbator';

cols = {pheno, cols{~phenocol}};

% discretize phenotype: (indicator of someone that has an exacerbation

% while on more steroids that usual)

data(:,1) = data(:,1) > 0;

% set discrete indicator

disc = [true, false(1,length(cols)-1)];

RACE = 'race';

racecol = find(strcmpi(RACE,cols));

disc(racecol) = true;

% normalize all other datacolumns:

fprintf(1,'Normalizing continuous data columns\n');

for i=1:length(disc)

if (~disc(i))

data(:,i) = (data(:,i) - mean(data(:,i))) / std(data(:,i));

end

end

The following block of code initializes Bayesian priors and then computes Bayes Factors of all variables with the command

>> BFs = BayesFactorScore(data,cols,pheno,priorPrecision);

And then reduces the dataset down to a manageable number of informative variables by limiting further investigation to variables with log Bayes Factor > 3. A Bayes Factor is a Bayesian likelihood ratio test and is useful for determining the strength of association of a variable with the phenotype of interest. We then split the data into two halves, a training and a testing half. In general analysis, a random split is advised, however for purposes of reproducibility, we deterministically split the data into the first half of the examples and the second half of the examples.

%% now test some bayesian stuff:

priorPrecision.nu = 50;

priorPrecision.alpha = 50;

priorPrecision.sigma2 = 1;

priorPrecision.maxParents = 2;

% test bayes factors: BFScore

fprintf(1,'Computing Bayes Factors for all %d variables\n',length(cols));

BFs = BayesFactorScore(data,cols,pheno,priorPrecision);

fprintf(1,'Found %d variables with positive Bayes Factor\n',sum(BFs > 0));

% finds ~250 variables.

keep = BFs > 3;

% keep race in the mix:

racecol = find(strcmpi(RACE,cols));

keep(racecol) = true;

keep(1) = true;

cols = cols(keep);

data = data(:,keep);

% normally, a random split is advised. However, for purposes of

% reproducibility, just take the first half:

%rvec = rand(1,length(data(:,1)));

splitvec = zeros(length(data(:,1)),1);

splitvec(length(splitvec)/2+1:end) = 1;

traindata = data(splitvec > 0.5,:);

testdata = data(splitvec < 0.5,:);

Next we perform actual Bayes Net learning. We start by learning a pheno-centric network using the command

>> PhenoCentricBN = LearnPhenoCentric(data, cols, pheno, priorPrecision, BFTHRESH, true, disc);

This function takes a data set (data and column names), a phenotype to predict, the Bayesian priors, and builds a pheno-centric network starting with that phenotype and then adding all additional edges with greater than BFTHRESH Bayesian likelihood that are within the Markov blanket of the phenotype. LearnPhenoCentric() returns a BayesNet class object that contains the network structure, network data, and parameters of the Bayesian network discovered. The following block of code performs this task. The command

>> PhenoCentricBN.WriteToGML('GEO_test_phenocentric_full');

Will write out a GraphML version of the network to a text file, GEO_test_phenocentric_full.graphml, that can then be loaded into network visualization software such as Cytoscape. Open that file now to see the pheno-centric network discovered.

%% Try building a Pheno-Centric BN:

% phenocentric search

BFTHRESH = 0;

tic;

fprintf(1,'Building PhenoCentric BN over %d variables\n',length(cols));

PhenoCentricBN = LearnPhenoCentric(data, cols, pheno, priorPrecision, BFTHRESH, true, disc);

t = toc;

fprintf(1,'Done Building PhenoCentric BN in %5.2f seconds\n',t);

PhenoCentricBN.WriteToGML('GEO_test_phenocentric_full');

Similarly, we may want to use the other learning algorithms provided with CGBayesNets to build Bayesian networks from this data. The following block of code executes a K2 search strategy on these data.

%% K2 search

tic;

fprintf(1,'Building K2 BN over %d variables\n',length(cols));

[K2MBnet, K2FullBN] = LearnStructure(data, cols, pheno, priorPrecision, 'K2NetTest', true);

t = toc;

fprintf(1,'Done Building K2 BN in %5.2f seconds\n',t);

K2FullBN.WriteToGML('GEO_test_K2_full');

Finally we try learning the third type of Bayesian network, the full network:

%% exhaustive search

BFTHRESH = 0;

fprintf(1,'Building Exhaustive BN over %d variables\n',length(cols));

tic;

ExhaustiveBN = FullBNLearn(data, cols, pheno, BFTHRESH, 'ExhaustiveBN', priorPrecision, disc);

t = toc;

fprintf(1,'Done Building Exhaustive BN in %5.2f seconds\n',t);

ExhaustiveBN.WriteToGML('GEO_test_Exhaustive_full');

These networks have been built over the whole dataset (not merely training or testing portions of the data). The next block of code will use the split data separately, first learning a network upon the training data half, and then testing the performance upon the testing data half.

%% ModelLearnAndTest

% should test this with many discrete vars (in TestSuite.m):

algorithm = 1;

verbose = true;

[auc, numnodes, testauc, model, classacc, testclassacc] = ...

ModelLearnAndTest(traindata, cols, testdata, cols, pheno, priorPrecision, ...

'MLearnAndTest1', verbose, {}, algorithm);

The above commands provide the output:

Start learning Bayesian network structure!

!Done learning Bayesian network!

Identified 23 variables for predicting Steroid Exacerbator.

Learning Network Parameters

Prediction on Training Data:

Prediction on Testing Data:

Prediction AUC: 1 [NaN,NaN], and convex-hull AUC: 1 [NaN,NaN].

Prediction AUC: 0.77318 [0.65495,0.89141], and convex-hull AUC: 0.8208 [0.70257,0.93903].

The ModelLearnAndTest() command takes two datasets, one for learning and one for testing, and an indicator variable for which learning algorithm to use (algorithm = 1 here indicates using the K2 network search procedure). This provides output in Area Under receiver operator characteristic Curve (AUC) where 50% is equivalent to random guessing and 100% indicates perfect classification of cases and controls. The output line:

Prediction AUC: 1 [NaN,NaN], and convex-hull AUC: 1 [NaN,NaN].

Indicates that an AUC of 1 or 100% is achieved in the training data; or perfect classification. The testing data performance is given in the next line:

Prediction AUC: 0.77318 [0.65495,0.89141], and convex-hull AUC: 0.8208 [0.70257,0.93903].

Here, the AUC is given first with its raw value and in brackets the 95% confidence interval for this quantity; and then the convex-hull of the AUC is given with confidence intervals. Classifiers can always be constructed to obtain the prediction indicated by their convex-hull AUC, and thus, this is the preferred measure. The Bayesian network is output in the variable “model” and a command like:

>> model.WriteToGML('GEO_test_modellearnandtest');

Can be used to obtain an output file of the network that may be visualized in Cytoscape.

The next block of commands uses a higher Bayes Factor filter to build Bayes Nets with only the most informative variables, illustrating the use of the command BFFilterBNLearn().

%% BFFilterBNLearn()

BFTHRESH = 6;

algorithm = 1;

[auc,MBNet] = BFFilterBNLearn(data, cols, pheno, algorithm, BFTHRESH, verbose, priorPrecision);

algorithm = 2;

[auc,MBNet] = BFFilterBNLearn(data, cols, pheno, algorithm, BFTHRESH, verbose, priorPrecision);

algorithm = 3;

[auc,MBNet] = BFFilterBNLearn(data, cols, pheno, algorithm, BFTHRESH, verbose, priorPrecision);

These commands take the usual input plus an indicator for which learning algorithm to use (1 = K2, 2 = pheno-centric, 3 = exhaustive), and a Bayes Factor threshold for inclusion of any variables into the model. All variables considered for potential inclusion in the network by BFFilterBNLearn() must have Bayes Factor > BFTHRESH for association with the phenotype. AUC output provided by this function is for the whole dataset provided.

%% Run additional Demonstration functions in DemoAnalysis()

% Test CV

% Test Bootstrapping

tdatas = {traindata,testdata};

tcols = {cols,cols};

DemoAnalysis(tdatas, tcols, pheno, 'GeoTestDemo', priorPrecision, 5, 20);

This command calls the DemoAnalysis() function which performs a comparison of the three different learning algorithms on the testing dataset using cross validation and bootstrapping. This uses CVModelLearnEx() and CVParamTest() to perform cross validation. The first function performs cross validation over model structure, while the second performs cross validation over model parameters only. Within the file DemoAnalysis() there are sections of code that utilize these cross validation routines:

%% do K2

[K2MBnet, K2FullBN] = LearnStructure(tdatas{1}, tcols{1}, pheno, priorPrecision, ...

[analysis_title,'K2'], verbose);

K2FullBN.WriteToGML([analysis_title,'K2_full']);

K2MBnet.WriteToGML([analysis_title,'K2_MB']);

auc_k2 = BNLearnAndTest(K2MBnet);

if (TESTING)

tauc_k2 = BNLearnAndTest(K2MBnet, tdatas{2},tcols{2});

end

In the above section, LearnStructure() is called to learn a network using K2 from only the training data. This returns two Bayesian Networks: the full Bayes Net over all the variables, K2FullBN; and the Markov blanket of the BN, K2MBnet, which is used for prediction. Both of these are then written to GraphML files with WriteToGML(). BNLearnAndTest() is a function that takes a BayesNet class object and trains the parameters on the data provided in that class object, and then tests the predictive performance of that object on the phenotype using either the provided test data (BNLearnAndTest called with three arguments) or on the data contained in the BayesNet class object (BNLearnAndTest called with just one argument).

%% do CV, params only:

fprintf(1,'Do CV on paramters from K2 network\n');

folds = 5;

[auc_k2cvparams, ~] = CVParamTest(K2MBnet, folds, '', verbose, false);

fprintf('total K2 network CV (params) AUC : %2.5f\n',100 * auc_k2cvparams);

The Markov blanket BayesNet object is then used in CVParamTest() to obtain an estimate of the predictive accuracy on unseen data. Compare auc_k2cv to auc_k2cvparams – this is the estimates of performance on unseen data using the two types of cross validation.

%% do bootstrapping to build a K2 network:

algorithm = 1;

fprintf(1,'Do Bootstrap building of K2 network\n');

BootsAdjMat = BootstrapLearn(tdatas{1}, tcols{1}, pheno, priorPrecision, ...

bootslimit, algorithm, verbose);

if (TESTING)

[k2_bsAUCs, k2_bsAUCs_test, k2_bsnumnodes] = BootsNetAddEdgesCVandTest(BootsAdjMat, ...

tdatas{1}, tcols{1}, tdatas{2}, tcols{2}, priorPrecision, pheno, nodelimit);

else

[k2_bsAUCs, k2_bsAUCs_test, k2_bsnumnodes] = BootsNetAddEdgesCV(BootsAdjMat, ...

tdatas{1}, tcols{1}, priorPrecision, pheno, nodelimit);

end

We then demonstrate code that performs bootstrapping sampling of the training data and builds different networks for each of those bootstrap realizations of the data, above. BootstrapLearn() takes a dataset, the number of bootstrap samples to perform, and the network search algorithm to use (algorithm = 1 for K2). This returns an adjacency matrix over all the variables in the dataset with fractional edge frequencies relating the relative occurrences of particular edges across the sample of bootstrap networks. We then use this as input into either BootsNetAddEdgesCV() or BootsNetAddEdgesCVandTest() to obtain cross-validation estimates of predictive performance on networks composed of the most frequently occurring N variables where N goes from 1 to NODELIMIT. These functions iteratively add the most frequent edge from the frequency matrix until a network of N nodes in the Markov blanket of the phenotype is achieved. With each network, cross validation is performed.

We repeat the bootstrap analysis above for each of the three learning algorithms, and an additional network identification method: naïve Bayes networks. Naïve Bayes networks have no edges except from the phenotype to other nodes, and are termed “naïve” because they assume no interactions among any of the predictors in the network. Finally all cross-validation performance and testing performance is shown in one graph, shown here in Figure S2. This figure shows the increasing performance of each network search strategy as more nodes are included in the network: that the K2 algorithm has the best performance in cross-validation but the naïve bayes networks have the best performance in the testing set.

To return to the code in TestDiffEx.m, we next demonstrate the use of the ModelLearnAndTest() function.

%% test ModelLearnAndTest

verbose = true;

algorithm = 1;

[auc, numnodes, testauc, BNModel, classacc, testclassacc] = ...

ModelLearnAndTest(traindata, cols, testdata, cols, pheno, priorPrecision, ...

'MLAT_GeoTest', verbose, {}, algorithm);

algorithm = 2;

[auc, numnodes, testauc, BNModel, classacc, testclassacc] = ...

ModelLearnAndTest(traindata, cols, testdata, cols, pheno, priorPrecision, ...

'MLAT_GeoTest', verbose, {}, algorithm);

algorithm = 3;

[auc, numnodes, testauc, BNModel, classacc, testclassacc] = ...

ModelLearnAndTest(traindata, cols, testdata, cols, pheno, priorPrecision, ...

'MLAT_GeoTest', verbose, {}, algorithm);

The ModelLearnAndTest() function combines tasks accomplished by several of the other CGBayesNets function: it learns a Bayesian Network over the training data using one of the three learning algorithms, then test the performance of that network on the testing data.

For comparison purposes, we have included a simple routine to build logistic regression models from the same types of datasets as we build Bayesian Networks. In principle, this allows a comparison of different predictive modeling strategies; although logistic regression is not the focus of CGBayesNets.

%% LogFitAndTest

verbose = true;

[auc, good, numvars, testauc, model, classacc, testclassacc] = ...

LogFitAndTest(traindata, cols, testdata, cols , pheno, verbose);

Executing this portion of the code in TestDiffEx.m results in warnings being output by MATLAB. These indicate that there are too many predictors to make a linear model, and if further investigation into logistic models is warranted, we suggest reducing the number of data columns and rerunning LogFitAndTest().

%% RandomNetworkPVal()

fprintf(1,' --- Testing capacity for overfitting using RandomNetworkPVal ---\n');

algorithm = 1;

numsims = 10;

[tpval] = RandomNetworkPVal(priorPrecision, data, cols, pheno, numsims, algorithm);

fprintf(1,'Empirical p-value from label permutation testing: %f\n',tpval);

A different kind of test can be performed with RandomNetworkPVal(). This function does NUMSIMS label permutations of the dataset, and at each one, learns a network to predict the permuted dataset. It then measures this prediction in AUC and thus generates a null distribution of AUC values for prediction of permuted phenotypes using the variables given. The true network’s prediction of the true phenotype is then compared to this null distribution to return an empirical p-value. This provides a rough measure of the capacity for overfitting – how powerful the learning method is compared to how much noise there is in the dataset; in this example the p-value is zero, meaning all 10 of the permuted networks had worse prediction than the true network. We have used numsims = 10 here for computational expediency, but for robust estimation of the null distribution we recommend running with 100 or 1000 permutations.

To do a more thorough investigation into the efficacy of bootstrapping in this dataset, we use the commands BootstrapLearn(), ThreshBootNetAndTest(), and NEdgesFromBoots().

%% ThreshBootNetAndTest()

% do some phenocentric boots nets:

algorithm = 2; % pheno-centric search

verbose = true;

nboots = 25;

bootsadjmat = BootstrapLearn(traindata, cols, pheno, priorPrecision, nboots, ...

algorithm, verbose);

thresh = 0.5;

convexhullAUC = ThreshBootNetAndTest(bootsadjmat, traindata, cols, testdata, ...

cols, pheno, priorPrecision, verbose, thresh);

fprintf(1,' --- test a 10-node Bootstrap Network \n');

nnodes = 10;

[BN, MBnet] = NEdgesFromBoots(bootsadjmat, traindata, cols, pheno, nnodes);

MBnet.priorPrecision = priorPrecision;

auc = BNLearnAndTest(MBnet, traindata, cols);

auc = BNLearnAndTest(MBnet, testdata, cols);

fprintf(1,'\t 10-node Bootstrap Network AUC : %2.1f \n',auc * 100);

MBnet.WriteToGML('GEO_test_10nodePhenoCentricBoots');

fprintf(1,' --- test a 25-node Bootstrap Network \n');

nnodes = 25;

[BN, MBnet] = NEdgesFromBoots(bootsadjmat, traindata, cols, pheno, nnodes);

MBnet.priorPrecision = priorPrecision;

auc = BNLearnAndTest(MBnet, traindata, cols);

auc = BNLearnAndTest(MBnet, testdata, cols);

fprintf(1,'\t 25-node Bootstrap Network AUC : %2.1f \n',auc * 100);

MBnet.WriteToGML('GEO_test_25nodePhenoCentricBoots');

fprintf(1,' --- test a 100-node Bootstrap Network \n');

nnodes = 100;

[BN, MBnet] = NEdgesFromBoots(bootsadjmat, traindata, cols, pheno, nnodes);

MBnet.priorPrecision = priorPrecision;

auc = BNLearnAndTest(MBnet, traindata, cols);

auc = BNLearnAndTest(MBnet, testdata, cols);

fprintf(1,'\t 100-node Bootstrap Network AUC : %2.1f \n',auc * 100);

MBnet.WriteToGML('GEO_test_100nodePhenoCentricBoots');

The above section of code first conducts bootstrapping with BootstrapLearn() doing 25 bootstrap realizations of the dataset and making a pheno-centric network for each of those. Once we have the edge-frequency matrix from that computation, we have two methods of constructing consensus networks. The first is to use a threshold that indicates which edges to include in the consensus network: include all edges present in at least 50% (for example) of the bootstrap networks. This is accomplished with ThreshBootNetAndTest(). This returns an AUC of the resulting network. The other method is to include the N most frequently occurring edges, for some value of N. This is accomplished with NEdgesFromBoots(), which we invoke three times using values of NNODES = 10, 25, and 100. These generate three different networks, with the specified number of nodes. For each of these networks, we test their performance on the training and testing datasets using the BNLearnAndTest() function.

From the numerous analyses demonstrated here, we can see that models with many predictors that are similar or nearly naïve-Bayes perform very well in both the training and test portions of the expression data. In particular, the final pheno-centric model generated from the 100-most frequent nodes appearing in bootstrap realizations of the test set, show in file GEO_test_100nodePHenoCentricBoots.graphml, is nearly a naïve-bayes model, and achieves 98.7% AUC in training and 87.1% AUC in the test dataset.

This sequence of analysis demonstrates the utility of CGBayesNets ability to generate and test predictive models in genomic datasets. The final model may be suggestive of a set of genes who’s expression is disregulated in asthma exacerbation; these may be useful for further biological inquiry or as a predictive model of individuals at risk for greater asthma exacerbations.

**Section 4. Illustrative Discretization Example**

We view the main differences between CGBayesNets and other existing free Bayesian network methods to be one of available features: CGBayesNets performs inference with mixed continuous and discrete networks; while other packages do not. To make this difference more concrete, we provide a simple example. We generated a network containing 5 discrete and 15 continuous nodes, and simulated data from that according to the randomly-chosen conditional distributions of each node. The data is contained in two files, both available with the CGBayesNets package, discretization/data_gen2_train.csv and discretization/data_gen2_test.csv. It is a dataset with 25 training examples and 25 testing examples, which is similar to modern genomic datasets in that the number of cases and controls is equal to (or, frequently, far less than) the number of variables considered for inclusion in the network model. One discrete node is designated the phenotype node, and is the one that we wished to predict based on the other nodes. Fourteen of the 20 nodes are in the Markov blanket of the phenotype, making the network quite dense for the small amount of data provided. We tested one method that relies on discretizing continuous variables, Weka 3.6.9, and one method that did not discretize continuous data while still including some inference capabilities, BNfinder 2.0, alongside CGBayesNets on this dataset.

Results are shown in Table 1. We first built networks using each method on the training data, then tried to predict the value of the phenotype node on each example in the testing dataset. We performed this analysis for the original data and for the data after discretization, where discretization was performed on training and testing data separately, using the default discretization method used in Weka 3.6.9. Since BNfinder 2.0 requires node-parent constraints, we provided constraints consistent with the topological ordering of nodes in the true network, and also the reverse of that order. The true ordering constraints (in Table 1, “BNfinder 2.0 (K2)”) resulted in models that had zero parents of the phenotype and that obtained zero predictive value (AUC = 50%). The opposite ordering actually resulted in good prediction (“BNfinder 2.0 (reverse K2)”) with the original data, finding several parents of the phenotype node. In the true network the phenotype node has several children, but no parents; this is generally a useful way to build predictive models, since prediction of a node with no parents and many children results in a naïve-Bayes classifier, which can be a very effective model [[4](#_ENREF_4)], while predicting a node with many parents and no children results in data fragmentation and overfitting. We speculate that nodes without parents cannot be predicted in BNfinder 2.0. Using the reverse-K2 ordering, BNfinder 2.0 had poor prediction with the discretized data; we speculate the inability to model the true dependencies in the data lead to this issue. Weka 3.6.9 requires discretization and so has no score on the original data. Weka’s performance on the discretized data is poor; this is evidently due to overfitting, since Weka 3.6.9 identified all 20 of the nodes as predictors of the phenotype. In each of the original and discretized data, CGBayesNets achieves equal or greater predictive performance than the other two methods.

This simple example was chosen as an illustration of a classification task including discrete and continuous variables that is ill-suited to the discretization method provided by default in Weka 3.6.9: discretizing continuous variables into 10 bins of equal size across the observed domain of that variable. To make a broader comparison, we simulated an additional 10 networks as described above, and tested each method upon data from those networks; the average performance is reported in Table 1. The average performances show much the same trends; if not as extreme: discretization results in poorer performance. It is a truism that discretization of continuous attributes can result in information loss, particularly if one chooses to use a method inappropriate for the dataset, and this in turn may lead to poor prediction. For more data on this particular case, see supplemental Figure S3, where we simulate many such datasets, and show that the performance of this discretization method is poor when the sample size is small compared to the number of variables, but good when the sample size is large. We also stress that there are many types of discretization schemes [[5](#_ENREF_5),[6](#_ENREF_6)], some of which are quite complicated and computationally expensive, and a thorough analysis of the pros and cons of each is beyond the scope of this paper. It is our main goal to provide exact methods of employing Bayesian networks in the presences of continuous variables without discretization, for those applications where discretization is inappropriate or deemed ineffective.

**Section 5. Theoretical Foundation**

A BN represents a joint distribution over a set of variables as a product of conditionally-independent probability distributions for each variable, where each variable is conditionally independent of each other variable, given the values of its parents in the BN. In a CGBN, discrete and continuous data are mixed, modeling discrete nodes as conditionally independent probability distributions dependant on the values of their discrete parents, and modeling continuous nodes as conditionally independent Gaussian distributions linearly dependent upon their Gaussian parents and with parameters conditioned on the values of the discrete parents. This model results in a multivariate normal mixture density over the domain of all variables. If the CGBN has a directed acyclic graph G over discrete variables Δ and continuous variables Ψ, where π(X) is the (possibly empty) set of parents of variable X according to G, and there is a set of conditional probability distributions P over Δ, and a set of conditional linear Gaussian density functions F over Ψ, then the multivariate normal mixture density over all variables [[7](#_ENREF_7)] is:

$$P\left( \Delta\right)f\left( \Psi| \Delta\right)=\prod_{x\in\Delta} P\left( x | \pi\left( x \right) \right)\prod_{y\in\Psi} f\left( y | \pi\left( y \right) \right)$$

When π(X) is empty, P(X) and f(Y) are just (unconditional) probability or density functions, respectively. Each continuous variable y, with U = π(y) in Δ, V = π(y) in Ψ, is normally distributed with parameters dependent upon the discrete parents of y:

$y|u,v \sim N\left( \alpha\left( u \right)+\beta\left( u \right)v,\sigma^{2}\left( u \right) \right)$.

To determine the best network model of the data, we compute the marginal likelihood of candidate network structures, conditioned upon the data, and choose the network model that maximizes the marginal likelihood. The posterior probability of the Bayesian network model G, given the data D, is p(G|D)p(D), and we use Bayes’ theorem to equate p(G|D)p(D) = p(D|G)p(G), or :

$p(G│D)\propto p(D│G)p\left( D \right)$,

Where p(G) is the prior probability of a network model and p(D) is the prior probability of the data, and p(D|G) is the marginal likelihood:

$p\left( D | G \right)=\int p\left( D | \theta,G \right)p\left( \theta| G \right)d\theta$.

Here p(D| θ,G) is the likelihood of the data given the network G and distribution parameters θ, and p(θ|G) is the prior density of the parameters θ. The marginal likelihood p(D|G) is computed by averaging out the distribution parameters θ from the likelihood function, p(D|G, θ). The Bayesian network semantics provides a decomposition of the likelihood as follows, for a given set of distribution parameters θ, a dataset D of size |D| = d, variables y_i_ in I = (Δ union Ψ) realizing values y_ik_ in {y_i1_, y_i2_, … y_id_} in D, given parents π(y_i_) take values u_ik_ when y_i_ takes value y_ik_:

$$p\left( D | G,\theta\right)\propto\left[ \prod_{i\in I} \prod_{k=1}^{d} p\left( y_{ik} | \pi\left( y_{ik} \right)=u_{ik},\theta_{ik} \right) \right]$$

where p(y_ik_| π(y_i_),θ_ik_) is the probability of y_i_ having value y_ik_ in D with parent values u_ik_ and distribution parameters θ. Distribution parameters for discrete nodes are modeled with Dirichlet priors, priors for Gaussian nodes are described below. In the discrete case, we denote by |y_i_| and |π(y_i_)| the number of different values that y_i_ and π(y_i_) can assume, respectively; then the discrete nodes have (joint) likelihood

$$P\left( \Delta| \theta\right)=\prod_{i\in\Delta} \prod_{j}^{\left| \pi\left( y_{i} \right) \right|} \frac{\Gamma\left( \alpha_{ij} \right)}{\Gamma\left( \alpha_{ij}+n_{ij} \right)}\prod_{k}^{\left| y_{i} \right|} \frac{\Gamma\left( \alpha_{ijk}+n_{ijk} \right)}{\Gamma\left( \alpha_{ijk} \right)}$$

where n_ijk_ is the number of data points satisfying y_i_ = k for π(y_i_) in configuration j, and α_ijk_ is the hyper parameter of the Dirichlet distribution indicating a prior assumed sample size. Γ(.) denotes the gamma function. Continuous nodes y_i_ have Gaussian distributions with a mean that is a linear function of its continuous parents and depending on its discrete parents, with a conditional variance σ^2^_ij_ = 1/τ_ij_. The joint likelihood of the continuous nodes is then

$$p\left( \Psi| \theta\right)=\prod_{i\in\Psi} \left( \frac{\tau_{ij}}{2\pi} \right)^{\frac{n}{2}}e^{\left[ \left( -\frac{\tau_{ij}}{2} \right)\left( y_{ik}-X_{i}\beta_{ij} \right)^{T}\left( y_{ik}-X_{i}\beta_{ij} \right) \right]}$$

with X_ij_ the values of continuous parents of y_i_ in case k, and β_ij_ the vector of regression parameters given discrete parents of y_i_ = j. We then follow Sebastiani *et al.*[[8](#_ENREF_8)] and use a Gamma prior distribution for τ and a conditional multivariate Gaussian prior density on regression parameters β. Thus,

$$\tau_{ij}\sim\Gamma\left( \alpha_{i1},\alpha_{i2} \right), p\left( \tau\right)=\frac{\tau_{ij}^{\alpha_{i1}-1}e^{{-\tau_{ij}}/{\alpha_{i2}}}}{{\alpha_{i2}}^{\alpha_{i1}}\Gamma\left( \alpha_{i1} \right)}$$

And β is described by

$$\beta_{ij}|\tau_{ij}\sim N\left( \beta_{ij0},\left( \tau_{ij}I \right)^{-1} \right)$$

For the identity matrix I, and β_ij0_ = E(β_ij_ | τ_ij_). The above equations represent the main semantics of CGBayesNets. For more detail, we refer the interested reader to Chang and Ramoni [[9](#_ENREF_9)].

Supplemental References

1. Xia J, Mandal R, Sinelnikov IV, Broadhurst D, Wishart DS (2012) MetaboAnalyst 2.0--a comprehensive server for metabolomic data analysis. Nucleic acids research 40: W127-133.

2. DeLong ER, DeLong DM, Clarke-Pearson DL (1988) Comparing the areas under two or more correlated receiver operating characteristic curves: a nonparametric approach. Biometrics 44: 837-845.

3. Bjornsdottir US, Holgate ST, Reddy PS, Hill AA, McKee CM, et al. (2011) Pathways activated during human asthma exacerbation as revealed by gene expression patterns in blood. PLoS One 6: e21902.

4. Domingos P, Pazzani M (1997) On the optimality of the simple Bayesian classifier under zero-one loss. Machine Learning 29: 103-130.

5. Dougherty J, Kohavi R, Sahami M. Supervised and unsupervised discretization of continuous features; 1995. Morgan Kaufmann Publishers, Inc. pp. 194-202.

6. Yang Y, Webb GI. A comparative study of discretization methods for naive-bayes classifiers; 2002. Citeseer.

7. Madsen AL (2008) Belief Update in CLG Bayesian Networks With Lazy Propagation. Int J Approx Reasoning 49: 503-521.

8. Sebastiani P, Abad M, Ramoni MF (2005) Bayesian Networks for Genomic Analysis. In: Dougherty ER, Shmulevich I, Chen J, Wang ZJ, editors. Genomic Signal Processing and Statistics. pp. 281-320.

9. Chang HH, Ramoni MF. Robust cross-race gene expression analysis; 2009 April 19-24; Taipei, Taiwan. pp. 505-508.

Table S1. Results of four Bayesian networks from bootstrapping on ‘Human_cachexia.csv’.

| Model | Total Nodes | AUC |
| --- | --- | --- |
| A | 2 | 82.9% |
| B | 3 | 77.7% |
| C | 4 | 83.5% |
| D | 8 | 86.8% |

Table S1. Predictive performance of four different networks on predicting muscle loss in the human cachexia metabolomics dataset. Models are those shown in Figure S2 (a, b, c, and d). The threshold column reports the minimum proportion of bootstrap realization networks that must include the edge for the edge to appear in the consensus network (see Figure S2). Total nodes reports the size of the network generated by the specific threshold. And AUC reports the convex hull of the Area Under receiver operator characteristic Curve, which measures prediction at various sensitivity and specificity combinations.

Figure Captions.

Figure S1. Bayesian Networks of Cachexia.

These networks are formed by running CGBayesNets bootstrapping routine on the human cachexia dataset from ([http://www.metaboanalyst.ca/MetaboAnalyst/faces/Home.jsp ‘human_cachexia.csv](http://www.metaboanalyst.ca/MetaboAnalyst/faces/Home.jsp%20'human_cachexia.csv)’). Each network shows the Markov blanket of the phenotype of interest (“Muscle loss”), which are those nodes necessary to predict muscle loss. Arrows between nodes indicate statistical dependence of the child node on the parent node(s), and do not represent causality. Networks are learned on 25 bootstrap realizations of the data, and those shown are consensus networks including the two (a), three (b), four (c), and eight (d) most frequently included edges in the bootstrap networks. Performance of these networks is given in Table 2. Images generated and formatted with the yEd program (yWorks).

Figure S2. Comparison of four different network search algorithms on training and test data.

Training performance is measured with five-fold cross-validation. For each of four search algorithms (K2, Pheno-Centric, Full-Exhaustive, and Naïve-Bayes) 5 bootstrap realizations of the training data were generated and a Bayesian network was learned for that realization. The x-axis represents using the most frequent N edges occurring in the population of bootstrap networks to create a consensus Bayesian network of at least N edges.

Figure S3. Comparison of prediction performance with continuous features vs. discretized features.

This graph shows the difference in predictive performance (measured by change in AUC predicting the phenotype node from training dataset to testing dataset) in a dataset including continuous variables and the same dataset after continuous datasets were discretized into 10 equal-sized bins. Each circle represents a different random network created on 25 nodes, each randomly chosen to be discrete or continuous. The size of the circle is proportional to the number of datapoints simulated from that network, N, ranging from 25 at the smallest circles to 200 at the largest circles. Experiments are ordered by increasing sample size (N), along the x-axis. The color of the circle represents the number of nodes in the Markov blanket of, and therefore required for prediction of, the phenotype node. The red line represents a regression of difference in predictive performance on the x-axis. This regression indicates that when the number of variables is similar to the sample size, performance is on average 13% worse after discretizing continuous variables; while the difference goes away when sample size far exceeds the number of variables. Experiments where there is no difference between continuous performance and discretized performance not included in this analysis.
